# Inflammatory Bowel Disease-Associated Spondyloarthritis

**DOI:** 10.3390/jcm15124680

**Published:** 2026-06-16

**Authors:** Edit Végh, Rebeka Falcsik, Nóra Bodnár, Szilvia Szamosi, Sándor Szántó, Gabriella Szűcs, Zoltán Szekanecz

**Affiliations:** 1 Department of Rheumatology, Faculty of Medicine, University of Debrecen, 4032 Debrecen, Hungary; vegh.edit@med.unideb.hu (E.V.); falcsik.rebeka@med.unideb.hu (R.F.); bodnar.nora@med.unideb.hu (N.B.); szamosi.szilvia@med.unideb.hu (S.S.); szanto.sandor@med.unideb.hu (S.S.); szucs.gabriella@med.unideb.hu (G.S.); 2 Department of Sports Medicine, Faculty of Medicine, University of Debrecen, 4032 Debrecen, Hungary

**Keywords:** spondyloarthritis, inflammatory bowel disease, SpA-IBD, Crohn’s disease, ulcerative colitis, microbiome, targeted therapy

## Abstract

Spondyloarthritis associated with inflammatory bowel diseases (IBD-SpA), also known as enteropathic arthritis, is an independent entity belonging to the spondyloarthritis (SpA) group. In recent years, a great amount of new data has been published regarding the pathogenesis and treatment of the disease. In this narrative review we present the main pathogenetic pathways along the gut–joint axis, the genetics of IBD and SpA, the role of environmental factors and that of the microbiome, as well as the main immunopathological processes including immune cells and inflammatory mediators. We cover the clinical picture, the specifics of axial and peripheral SpA-IBD, and briefly discuss the diagnostics. There are common options in the pharmacotherapy of SpA and IBD; however, some drugs that control arthritis (e.g., NSAIDs, IL-17 inhibitors) might not be suitable for the treatment of IBD. Based on the pathogenetic role of the microbiome, it is suggested that pharmacotherapy can be supplemented with non-pharmacological remedies, such as diet including the administration of short-chain fatty acids (SCFAs). Finally, we briefly look at the future that might include rational, even personalized treatment.

## 1. Introduction

Spondyloarthritis (SpA) associated with inflammatory bowel diseases (IBD), referred to as IBD-SpA in this paper, but also known as enteropathic arthritis (EA), is the association of SpA with either Crohn’s disease (CD) or ulcerative colitis (UC) [1]. IBD-SpA is a separate entity within the group of SpA that, according to the classical ESSG criteria, also includes ankylosing spondylitis (AS), psoriatic arthritis (PsA), and reactive arthritis [1,2]. Since then, SpA has been increasingly recognized as a disease continuum [3]. The currently valid ASAS classification criteria categorize SpA into axial and peripheral forms based on clinical presentation (Table 1) [3]. There is a debate between rheumatologists and gastroenterologists, whether IBD-SpA is SpA associated with IBD as an extraintestinal manifestation or IBD associated with SpA as an extraarticular involvement [4]. Usually IBD precedes SpA; however, the association of IBD and SpA requires early diagnosis and management by both medical disciplines [4]. We hereby present a narrative review on IBD-SpA using available relevant publications after a search of PubMed.

## 2. Epidemiology

There is a known close association between IBD and SpA. IBDs are associated with arthritis in approx. 20–40% of cases, while 20–30% of SpA patients have overt IBD. IBD-SpA has higher prevalence in women (67%) [1,5,6]. Within SpA, most literature data come from AS patient cohorts. IBD most frequently occurs within the first year after the diagnosis of AS [1,7]. The prevalence of peripheral arthritis, enthesitis and sacroiliitis in IBD are approx. 13%, 20% and 10%, respectively [1,5,6]. In a comparative evaluation of all extra-articular manifestations of SpA, anterior uveitis was the most common, followed by IBD and then psoriasis [8].

There is also evidence showing a close relationship between inflammatory activity in the joints and the intestine [9,10]. In AS, silent (subclinical) microscopic and/or macroscopic intestinal inflammation can be detected in approx. 60% of cases. Then clinical IBD develops within 5 years [11,12]. In SpA, CD is more common than UC; moreover, sacroiliitis (axSpA) and peripheral SpA develop somewhat more frequently in CD than in UC (Table 1) [1,12,13].

## 3. Etiopathogenesis

### 3.1. Genetic Factors

Genetic determinants including both HLA-B27 and non-HLA genes are crucial in the development of IBD-SpA (Table 1) [6,13,14,15]. First-degree relatives of SpA patients have a threefold risk of developing IBD and vice versa. The prevalence of HLA-B27 is slightly lower (50–70%) among IBD-SpA patients than in AS [14]. The pathogenic role of HLA-B27 including molecular mimicry and unfolded protein response (UPR) in SpA has been established [15] and will not be discussed here in more detail. Moreover, HLA-B27 transgenic rats develop a syndrome like IBD and SpA. However, the importance of external pathogens discussed later is indicated by the fact that these diseases do not develop under sterile conditions [5,6]. HLA-B27 positivity was independently associated with axSpA development in CD [1].

Among the non-HLA genes, those of protein tyrosine phosphatase 22 (*PTPN22*), interleukin 23 receptor (*IL23R*) and *CARD15/NOD2* are likely to play a role in both IBD and SpA [5,6,16]. Since the *PTPN22* and *IL23R* genes are primarily autoimmune, and *NOD2* is mainly autoinflammatory, this also demonstrates the mixed nature of IBD-SpA from a genetic perspective [17]. The role of *STAT3* gene variants has also been suggested in both IBD and SpA, confirming the probable involvement of the JAK-STAT system in disease etiopathogenesis [5,18].

### 3.2. The Role of the Microbiome and Other Risk Factors

The gut–joint axis, changes in the microbiome (dysbiosis) seem to be fundamentally important in the development of both IBDs and SpA [1,12,19]. In the composition of the normal human intestinal microbiome, the dominant flora is 30–50% Firmicutes (e.g., *Clostridium*, *Ruminococcus* species), 9–40% Bacteroidetes (e.g., *Bacteroides*, *Prevotella*, *Porphyromonas* species), and 1–13% Actinobacteria (e.g., *Collinsella* species) [1,12,20]. This symbiosis changes in inflammatory diseases when the ratio of some species increases, and the overall species heterogeneity decreases. For example, while ~600 species are found in the feces of healthy individuals, only ~400–450 species have been identified in SpA patients. In addition, the proliferation of commensal bacteria and increased permeability of the intestinal wall (“leaky gut”) are characteristic for IBD and SpA [1,21]. Increased expression of intestinal zonulin, dysbiosis and increased gut permeability have been associated with axSpA/AS [1,21]. Regarding dysbiosis, in SpA, the majority of the studies suggest that the key microbial player would be the *Ruminococcus* species (Firmicutes). For example, *R. gnavus* has been observed in the intestine at the expense of other species. The amount of *R. gnavus* was higher in patients with IBD-SpA compared to patients without IBD and was also correlated with BASDAI [1,20]. Other important microbes include *Mediterraneibacter gnavus* [1,21] and *Dialister* species (also Firmicutes) [20]. The amount of *Dialister* was also correlated with the disease activity of SpA (ASDAS) [20]. The Firmicutes/Bacteroidetes (F/B) ratio has been introduced to characterize the normal or dysbiotic gut microflora [20]. Less importantly, among bacterial species linking IBD and SpA, increased amounts of *Pseudomonas*, *Enterococcus*, *Lactobacillus*, *Bifidobacterium*, *Klebsiella* and *Proteus* species and, on the other hand, relatively reduced rates of *Faecalibacterium*, *Fusobacteria* and *Rosiburia* species are characteristic for both disease forms [12,13,21,22].

In a recent study, risk factors for developing SpA in IBD patients also included female sex (OR: 2.1 [95% CI: 1.0–4.3) and concomitant psoriasis (OR: 2.7 [95% CI: 1.0–6.8) [23].

### 3.3. The Development of Gut and Joint Inflammation

As a result of genetic background, intestinal dysbiosis and, consequently, “leaky gut”, pathogenic microbial antigens can stimulate mucosal immune-inflammatory cells. These cells including T_H_1-, T_H_17-, NK-, NK-T-, mast cells and neutrophils occurs in the gut with the suppression of T_H_2-, T_REG_- and B_REG_-cells. This is accompanied by increased production of pro-inflammatory cytokines, such as tumor necrosis factor α (TNF-α), IL-1, IL-6, IL-17, IL-22, and IL-23. The release of cells and cytokines into the bloodstream causes systemic inflammation including SpA [1,5,6,12,20]. Thus, among the immune cells T_H_17-cells, cytokines IL-23 and IL-17 play the most important role in the pathogenesis of both IBD and SpA [19,24,25]. To further determine the link between IBD and SpA-associated inflammation, recently, increased amounts of IL-17A and IL-23, in association with calprotectin, were found in the stool of axSpA patients [25].

Moreover, monocytes and lymphocytes belonging to the intestinal mucosal immune system, migrate to the spleen and then to the joint (homing). β1 and β7 integrins expressed by these migrating cells are highly involved in mucosal homing processes. These adhesion molecules are expressed in both the gut and the synovium. As discussed later, the cells, cytokines and integrins mentioned above serve as important targets for the therapy of IBD-SpA [5,12,13,19,26,27,28]. Already in the late 1990s, it was shown that mucosal leukocytes isolated from the intestinal wall of IBD patients adhere to high endothelial venules of the synovial tissue [28]. Later, in vivo studies also confirmed that IBD lymphocytes migrate from the intestine to the joint and this pathological “homing” can be inhibited by antibodies against individual adhesion molecules (e.g., α4, β1, β7 integrins) [26].

Although the basic inflammatory pathways have been characterized in connection with the appropriate genetic background in IBD-SpA, the exact pathomechanism of how arthritis is linked to intestinal inflammation is still not fully understood. According to one hypothesis, generalized inflammation in the joints develops in parallel or after the onset of subclinical and clinical intestinal inflammation. On the other hand, immune cells involved in mucosal inflammation recirculate between the gut and the joints. Finally, maybe most importantly, the role of the shared microbiome has also been hypothesized resulting in changes in the intestinal bacterial flora potentially triggering arthritis. Each hypothesis has its significance [5,12,13,19,20].

Synovial histopathology in IBD-SpA highly resembles that in other forms on SpA rather than RA. It is characterized by synovial lining hyperplasia, leukocyte infiltration with the predominance of T-cells, macrophages and plasma cells, as well as increased synovial angiogenesis with less pannus formation and erosions compared to RA [29,30,31,32].

## 4. Clinical Aspects and Diagnostics

In the clinical practice, IBD-SpA can be relatively easily distinguished from other inflammatory arthritides by looking for clinical signs and symptoms characteristic for SpA (Table 1). Traditionally, axial and peripheral joint involvement are to be investigated. In general, peripheral arthritis can be classified into clinical forms. Peripheral arthritis type 1 (PeA1) is characterized by oligoarthritis with the involvement of <5 joints, and by an asymmetric appearance, predominantly in the lower extremities and in larger joints. PeA1 usually follows the inflammatory activity of IBD and can be short-term and transient. In PeA2, one can expect a more symmetrical, polyarticular presentation, which is independent of IBD flares and can persist for years. In both types, arthritis is typically non-erosive and often only arthralgia is observed. Dactylitis and enthesitis occur less frequently compared to other types of peripheral arthritides [1,5,6,13].

With respect to axial involvement, sacroiliitis often occurs asymptomatically, which often does not progress to spondylitis. Characteristic axSpA/AS often precedes IBD and occurs independently of intestinal inflammation. Of course, axial and peripheral involvement can also occur simultaneously. Overall, any, either peripheral or axial SpA is associated more often with CD than with UC [5,6,13]. In conclusion, it is important to differentiate the characteristics of musculoskeletal manifestations and disease phenotype to predict the expected disease duration, course and optimal treatment [5,6,13].

Detailed history taking is crucial and various self-administered screening tools are available for the fast assessment of IBD-SpA [1,5,33]. The IBIS-Q and DETAIL tools consist of 14 and six questions, respectively. If the screening is positive, the patients should be examined by ultrasound and/or MRI/CT for SpA [1,33,34,35]. Questionnaire-based screening is only the first step, which should be followed by imaging, colonoscopy, video capsule endoscopy (VCE) and/or fecal calprotectin assessments [1,33,34,35].

Endoscopic procedures for detecting gastrointestinal inflammation are indispensable in the diagnosis of the disease, since intestinal symptoms can sometimes be subclinical [1,5,36]. With respect to intestinal involvement in IBD-SpA, patients with CD-SpA patients had less ileocecal involvement than CD patients and UC-SpA patients had more pancolitis than UC patients [1,37]. In the interesting SpACE Capsule study, VCE was used to prospectively detect and follow the development of CD in 64 SpA patients. VCE detected small bowel inflammation in more SpA patients (42%) than standard colonoscopy (11%). Thus, VCE might be more sensitive in detecting subclinical CD in SpA [1,36].

There is no gold-standard laboratory biomarker for IBD-SpA. One can use tests suitable to detect IBD and SpA anyway. Acute phase reactants are more indicative of IBD activity rather than that of SpA, particularly axSpA. Inflammatory leukocytes migrating to the sites of intestinal inflammation produce S100 proteins, of which S100A08 and S100A09 form the heterodimer called calprotectin. Calprotectin is a sensitive marker of intestinal inflammation, and its amounts may also increase in SpA [38]. The most relevant serum biomarkers of IBD-SpA include genetic markers (e.g., *HLAB27*, *NOD2*/*CARD15*), biomarkers of inflammatory activity (e.g., CRP, IL-6, IL-17, IL-23, matrix metalloproteinase 3 [MMP-3], serum amyloid A [SAA]), autoimmunity (e.g., p-ANCA, ASCA, anti-OmpC) and structural progression (e.g., DKK-1, sclerostin, tenascin, VEGF and serum calprotectin) [38].

Among imaging modalities, conventional X-rays only show advanced musculoskeletal bone changes. There are several characteristic morphological changes in SpA/AS, but their development occurs later during the disease course [39,40]. Therefore, MRI is the right choice for the early detection of sacroiliitis and spondylitis [5,6,12,13,40]. In the future, PET-CT that detects both intestinal and synovial inflammation might also be a diagnostic option [41].

Synovial biopsy and histopathology mostly serves research purposes. However, histology might be useful for the differentiation from other synovial pathologies [29,30,31,32].

Differential diagnosis of IBD-SpA should include assessment and exclusion of rheumatoid arthritis, osteoarthritis, reactive or infectious arthritis, fibromyalgia or other autoimmune diseases. Moreover, SpA may be associated not only with true IBD, but also with irritable bowel syndrome or NSAID-induced enteropathy [1,5].

## 5. Treatment from the Rheumatologist’s Viewpoint

There are several overlaps in the treatment of SpA and IBD and it requires cooperation between the two medical specialties during the management of IBD-SpA (Table 2 and Table 3). Targeted therapies are often the same due to the common pathogenetic pathways described above. Yet, during the therapy of IBD-SpA, we may encounter difficulties due to some differences in the drugs used in IBD or SpA. In general, rheumatologists follow treatment recommendations for peripheral SpA, primarily PsA and axSpA, while gastroenterologists use drugs for the treatment of CD and UC [1,5,6,12,42,43,44].

### 5.1. Non-Pharmacological Interventions

Regarding non-pharmacological treatment modalities, diet and restoration of microbial dysbiosis described above could also be targeted. Many studies with probiotics have been successful in various forms of arthritis [20,22]. On the other hand, short-chain fatty acids (SCFAs), such as acetate (C2), propionate (C3) and butyrate (C4), are metabolites produced by intestinal bacteria. SCFAs exert anti-inflammatory effects, thus inhibiting macrophage, T_H_1- and T_H_17-cell activation, as well as the production of pro-inflammatory cytokines. SCFAs also activate T_REG_ cells and enhance the production of the anti-inflammatory IL-10 [19,45]. In mice, 8 weeks of administration of C2, C3 and C4 SCFAs reduced the development of arthritis. This was mostly due to changes in the microbiome, as the SCFA diet restored the F/B ratio [45]. Large-scale controlled human studies of SCFA-containing diets in IMIDs should be conducted to determine the possible use of such diets in IBD-SpA [1,19,45]. Regarding other dietary approaches, low FODMAP (fermentable oligosaccharides, disaccharides, monosaccharides and polyols) and Mediterranean diets might be useful, although there have been no conclusive data on these diets [44]. Finally, there have been emerging advances in fecal microbiota transplantation (FMT) in IBD. The efficacy of FMT has been evaluated in several IMIDs including IBD or SpA/PsA. However, we do not have evidence that FMT would be clinically effective in IBD-SpA [44]. Yet, there is very limited evidence for the possible application of these interventions in human IBD-SpA.

### 5.2. Anti-Inflammatory Drugs

One would probably choose non-steroidal anti-inflammatory drugs (NSAIDs) in PeA1 and axial involvement (Table 3). However, long-term use of NSAIDs in IBD is contraindicated because they can cause mucosal damage in the entire gastrointestinal system. NSAIDs might impair the barrier function of the intestine by inhibiting prostaglandin production [46]. On the other hand, a systematic Cochrane analysis found two prospective studies examining the effect of COX-2 inhibitors (celecoxib, etoricoxib) used for 3 months on IBD. These coxibs did not significantly increase the risk of IBD relapse, and gastrointestinal side effects were reversible upon discontinuation of the COX-2 inhibitor [1,12,46,47]. The risk of IBD flares upon the use of non-selective NSAIDs tended to increase in a pooled analysis of 18 prospective and retrospective studies [46,48]. Overall, it seems that, in the absence of high-level evidence, non-selective NSAIDs can be used for short-term remission of IBD, while COX-2 inhibitors would be preferred in patients with increased risk of IBD activity (Table 2 and Table 3) [1,12,42,46].

In peripheral SpA, corticosteroids (CS) may be considered (Table 3). These compounds may also have a beneficial effect on the gut but may increase the risk of infection and intra-abdominal abscess formation if administered in higher doses [12,49].

### 5.3. Conventional Synthetic and Targeted DMARDs

According to recommendations, the use of conventional synthetic disease-modifying antirheumatic drugs (csDMARDs) should also be considered in the treatment of peripheral arthritis. Methotrexate (MTX) has been shown to be effective as a maintenance therapy for patients with CD but has little effect on UC (Table 2). Sulfasalazine (SSZ) can also be used in mild or moderate arthritis. SSZ is a possible choice for induction and maintenance therapy, especially in higher doses, in the treatment of UC (Table 2) [12,42,50].

**Table 3:** Comparison of the treatment of IBD, SpA and IBD-SpA.

	CD	UC	axSpA/AS	PsA	Axial IBD-SpA	Peripheral IBD-SpA	References
**Traditional NSAID**							[1,44,46]
**COX-2-selective NSAID**							[1,44,46]
**CS**							[1,43,44]
**MTX**							[1,6,23]
**SSZ**							[1,43]
**TNFi (mAbs)**							[1,6,12,23,43,44]
**TNFi (ETN)**							[1,23,44]
**IL-17i**							[1,6,12,19,25,51,52]
**IL-12/23i**							[1,23,43,44,52]
**IL-23i**							[1,12,23,44,52]
**antiintegrin** **(anti-α4β1, anti-α4β7)**							[1,19,26,27,42,44,53]
**JAKi**							[1,6,44,54]
**combinations (e.g., TNFi + csDMARD, TNFi + antiintegrin, IL-12/23i + TNFi, bDMARD + JAKi)**							[1,23,44,55,56]

Color codes: dark green: recommended; light green: possible; pink: recommended with caution; red: not advisable (contraindication); grey: no efficacy or efficacy not known. Abbreviations: AS, ankylosing spondylitis; axSpA, axial SpA; bDMARD, biologic DMARD; CD, Crohn’s disease; COX, cyclooxygenase; CS, corticosteroid; csDMARD, conventional synthetic DMARD; DMARD, disease-modifying antirheumatic drug; ETN, etanercept; i, inhibitor; IBD, inflammatory bowel disease; IL, interleukin; JAK, Janus kinase; mAb, monoclonal antibody; MTX, methotrexate; NSAID, non-steroidal anti-inflammatory drug; PsA, psoriatic arthritis; SpA, spondyloarthritis, SSZ, sulfasalazine; TNF, tumor necrosis factor; UC, ulcerative colitis.

Among biologic DMARDs (bDMARDs), TNF-α inhibitors (TNFis), primarily monoclonal antibodies, are considered gold standard treatment. Infliximab and adalimumab are the most used, as these bDMARDs have beneficial effects in both IBD and SpA (Table 2). We should not forget the practical fact that when using TNFi in IBD-SpA, we should follow a different dosing and treatment regimen than used by gastroenterologists in isolated IBD [1,5,6,12,42,44]. Certolizumab pegol and golimumab are only registered for CD and UC, respectively [5,42]. The TNF-α receptor fusion protein etanercept is of little efficacy in the treatment of IBD. Several hypotheses are known to explain this, one of which is that its apoptotic effect on T-cells in the lamina propria is insufficient. Etanercept blocks not only TNF-α but also TNF-β. The latter regulates T-cell-dependent IgA production, which in turn affects the composition of the gut microbiome. Etanercept can even cause flares or manifest subclinical inflammation in IBD, for example when patients with SpA were treated with this bDMARD [5,12,42].

Inhibition of the IL-12/23 pathway, which is known to be involved in the pathogenesis of IBD, SpA and thus IBD-SpA [1,13,52], and reduction in IL-17 levels have yielded contradictory results regarding the expected efficacy and safety [42,51,52]. Even if IL-17 might have a pathogenic role in intestinal inflammation [52], IL-17 inhibition, either with IL-17 inhibitors (IL-17i; secukinumab, ixekizumab, bimekizumab) or IL-17 receptor inhibitors (IL-17Ri; brodalumab), has been shown to be ineffective, in some cases even harmful in the treatment of IBD [1,42,51,52]. One explanation for this is that IL-17 has a protective effect on the intestinal mucosa. IL-17i causes an imbalance, dysregulation of mucosal cytokines, and a dominance of the T_H_1 pathway, which manifests as intestinal inflammation [42,51,52]. Another theory is that IL-17i may lead to the aggravation of IBD by altering the fungal microbiome in the colon (proliferation of Candida species). Indeed, IBD flares have been observed with IL-17i, which manifest as bleeding [42,51]. According to the recommendations, IL-17i/IL-17Ri, which is otherwise particularly effective in axSpA, is not used during active IBD; however, there is only a relative contraindication in cases of IBD in remission or subclinical IBD [42,51,52]. In the latter settings, after individual consideration of the benefit/risk ratio, an IL-17i can be used in appropriate cases and under close supervision in IBD and thus IBD-SpA [5,42,51,52].

The anti-IL-12/23 p40 inhibitor (IL-12/23i; ustekinumab) has proven effective in peripheral SpA, but not in axSpA [1,5,6]. This bDMARD can be used effectively in both CD and UC, but it is important to emphasize here that gastroenterologists use it in higher doses. IL-12/23i or IL-23 p19 inhibitors (IL-23i; e.g., guselkumab, risankizumab, mirikizumab, tildrakizumab), which seem to be variably effective in IBD, as well as psoriasis and PsA were not effective in axSpA [1,5,44]. Further investigations are needed to determine the value of IL-17i, IL-12/23i and IL-23i in IBD-SpA [1,5].

Integrin inhibitor bDMARDs (vedolizumab, natalizumab) can be used with good efficacy in IBD [1,5,44,57]. Vedolizumab is an intestinal-specific monoclonal antibody that binds to the α4β7 integrin and selectively inhibits leukocyte adhesion to the vascular MAdCAM-1 addressin, which is found in the intestinal endothelium and reduces inflammation locally [57]. In addition to its effect on IBD, its effect on arthralgia and arthritis is controversially based on a post hoc analysis of the GEMINI study [57]. A decreasing incidence was observed in CD patients, while it had no effect on musculoskeletal symptoms in UC [57]. Natalizumab inhibits the α4β1 integrin. It is used in Europe to treat multiple sclerosis, but it has been registered for CD in the US [53].

JAK inhibitors (JAKi) are targeted synthetic DMARDs (tsDMARDs) and they are also potential treatment options in both SpA and IBD. Among these agents, tofacitinib and upadacitinib have been approved in axSpA and peripheral SpA/PsA, upadacitinib has been registered in CD, while upadacitinib, tofacitinib and filgotinib in UC [1,44,54]. JAKi would be therefore well-suited for use in IBD-SpA (Table 2) [1,44,54].

It is also worth mentioning that there is a growing body of evidence about the combination of bDMARDs and JAKi, especially in the treatment of patients with therapy-refractory (difficult to treat) IBD or SpA [1,13,54,55,56]. Most data have been published on combinations of TNFi and either IL-12/23i or IL-23i, TNFi and vedolizumab, or JAKi and IL-12/23i. Combination treatments may be appropriate because in some patients the course of IBD and SpA do not change in parallel and sometimes these two conditions cannot be treated using a single DMARD. For example, IL-12/23i and IL-23i mostly affect IBD, in which case another drug (e.g., JAKi) should also be administered to control SpA [1,13,54,55,56]. Observational studies have shown that the use of such combined treatment strategies does not significantly increase the risk of adverse events, and they also seem to have a beneficial effect on the peripheral symptoms of SpA [1,13,54,55,56].

### 5.4. Treatment Strategy Recommendations

Potential tailored b/tsDMARD therapy in IBD-SpA is an important need for the patient and the practicing physician (Table 3) [1,5,24,43,44,58]. The basis of such treatment strategies relies on the complex inflammatory network of immune cells and cytokines linking the gut and joint described above [1,17,19,24,58]. Both IBD and SpA are mixed-pattern, immune-mediated inflammatory diseases (IMID) on the autoimmunity–autoinflammation landscape [17,59]. Most IMIDs respond well to TNFi, IL-12/23i and JAKi, while, as discussed above, IL-17i and IL-23i may have disparate effects and safety profile in IBD and SpAs [1,5,17,19,59]. With respect to IL-17i, disease activity of IBD is also an important point, as IL-17i might be used if IBD is in remission or only subclinical inflammation is present [19,51,52]. While a long time ago, when only TNFi agents were available, we thought that most IMIDs could be treated in a similar way, today we should move towards personalized, tailored treatment strategies (Table 3) [1,19,24,43,58]. Moreover, in known IBD patients the axial or peripheral nature may also determine the treatment of choice. In axSpA, first TNFi or JAKi should be considered followed by IL-12/23i. In mild forms of peripheral SpA, csDMARDs could be effective, while in moderate-to-severe forms, TNFi, JAKi, IL-12/23i or IL-23i could be tried [1,43,44].

There is no clear consensus and there have been no widely accepted official recommendations for the treatment of IBD-SpA. Recently, the Italian Group for the Study of Inflammatory Bowel Disease (IG-IBD) and Italian Society of Rheumatology (SIR) published their national recommendations based on a pseudo-Delphi consensus [60]. Altogether 34 statements and four therapeutic algorithms were formulated to be used as a reference guide for gastroenterologists and rheumatologists. In addition to consensus statements on diagnosis and classification of IBD-SpA, several treatment points were published in IBD-SpA with axial or peripheral involvement, or active or inactive IBD. “Red flags” for IBD included chronic diarrhea, rectal bleeding, perianal fistula or abscess, chronic abdominal pain, fever, anemia and weight loss. Similarly, special attention is needed in SpA in the case of chronic low back pain, enthesitis, dactylitis, peripheral active synovitis, psoriasis, anterior uveitis or chest pain. The four treatment algorithms are summarized in [60].

### 5.5. Outcome Measures and Prevention

The remission in IBD and SpA has been well characterized [3,38,60]. As described above, biopsy and histopathology of the gut and the synovium could serve both research and outcome purposes [29,30,31,32]. There is an important need for clinical, laboratory, endoscopic and other biomarkers that can predict the outcome of IBD-SpA and responses to therapy. These biomarkers include CRP, ESR, serum and fecal calprotectin and SAA [1,38,43].

Is there a possibility of prevention of IBD-SpA in patients with either IBD or SpA using bDMARDs? In a recent retrospective study, 507 CD and UC patients undergoing targeted therapy vs. csDMARDs were evaluated for the development of SpA. The use of bDMARD vs. csDMARD was highly protective throughout a 12-month period with an OR: 0.4 (95% CI: 0.2–0.8) [23].

### 5.6. Collaboration Between the Gastroenterologist and Rheumatologist During the Management of IBD-SpA

After all, a close collaboration between these two medical disciplines is crucial. As discussed above, IBD usually precedes SpA. Therefore, while the rheumatologist usually receives the patient with a known diagnosis of IBD, gastroenterologists should look for preliminary signs and symptoms of SpA. These include the known classification criteria of axSpA. Briefly, young age, male sex, inflammatory back pain during the night and maybe HLA-B27 testing could help to identify IBD-SpA [1,3,6,44].

## 6. Conclusions

In summary, IBD-SpA is an IMID with a typical genetic background and heterogeneous clinical phenotypes. The optimal diagnosis and treatment often pose a challenge to the rheumatologist. Regular professional consultations with a gastroenterologist are essential. It may be worth setting up an “IMID-team” to discuss more complex cases. We ourselves have recently implemented such a consultation system at the University of Debrecen. Of course, further research is needed to determine the optimal, more tailored treatment strategies that could benefit our IBD-SpA patients.

## Figures and Tables

**Table 1:** Characteristics of SpA in IBD-SpA.

	Peripheral		Axial	
**Type of arthritis**	PeA1	PeA2	isolated sacroiliitis	spondylitis
**Clinical characteristics**	asymmetric	symmetric	bilateral or unilateral	unilateral, often precedes IBD
**Joint involvement**	mono-oligoarthritis (<5 joints)	polyarthritis (≥5 joints)	asymptomatic or mild low back pain upon rest, less progressive	classical axSpA/AS
**Duration**	acute, self-limiting (<10 weeks)	chronic (months, years)		chronic (months, years)
**Relationship to IBD**	associated with IBD flare	independent of IBD flare		independent of IBD flare
**Genetics**	*HLADRB1, HLAB35*, *HLAB27*	*HLAB44*		*HLAB27*
**Prevalence in CD**	6%	4%		1.2–4%
**Prevalence in UC**	3.6%	2.5%		0.9–2%

Abbreviations: AS, ankylosing spondylitis; axSpA, axial SpA; CD, Crohn’s disease; HLA, human leukocyte antigen; IBD, inflammatory bowel disease; PeA, peripheral arthritis; SpA, spondylarthritis; UC, ulcerative colitis.

**Table 2:** Compounds for the possible treatment of IBD-SpA.

Compound	Target	Dose in SpA *	Dose in IBD	Use in IBD-SpA
COX-2-selective NSAIDs	COX-2	varies, based on the compound	varies; may be safer than traditional NSAIDs	COX-2-selective NSAIDs are safer
Methotrexate	folate, adenosine	15–25 mg/week PO or SC	15–25 mg/week PO or SC	Effective in peripheral SpA but not in axSpA
Sulfasalazin	anti-inflammatory	2–3 g/day PO	2–3 g/day PO	
Infliximab	TNF-α	5 mg/kg IV at weeks 0, 2, 6, then every 8 weeks	5 mg/kg IV at weeks 0, 2, 6, then every 8 weeks	Effective in both IBD and SpA
Adalimumab	TNF-α	40 mg SC every 2 weeks	160 mg SC at week 0, 80 mg SC at week 2, then 40 mg SC every 2 weeks	
Golimumab	TNF-α	50 mg SC every 4 weeks	200 mg SC at week 0, 100 mg SC at week 2 then 100 mg SC every 4 weeks	
Ustekinumab	IL-12/23 p40	45/90 mg SC at weeks 0, 4, then every 12 weeks	6 mg/kg IV, then 90 mg SC every 8–12 weeks	Effective in IBD and possibly in peripheral SpA but not in axSpA
Guselkumab	IL-23 p19	100 mg SC at weeks 0, 4, then every 8 weeks	200 mg SC at weeks 0, 4, then every 8 weeks	Effective in IBD and possibly in peripheral SpA but not in axSpA
Risankizumab	IL-23 p19	150 mg SC at weeks 0, 4, then every 12 weeks	600/1200 mg IV at weeks 0, 4, 8, then 180–360 mg SC at week 12 and then every 8 weeks	
Secukinumab	IL-17A			
Ixekizumab	IL-17A			
Bimekizumab	IL-17A/F			
Tofacitinib	JAK1/3	5 mg PO bid or 11 mg PO od	10 mg PO bid for 8 weeks, then 5 mg bid	Effective in IBD, peripheral SpA and axSpA
Upadacitinib	JAK1	15 mg PO od	45 mg PO od for 8 weeks, then 15/30 mg od	
Filgotinib	JAK1	200 mg PO od	200 mg PO od	

* No drugs are approved in the EU for the treatment of IBD-SpA. These drugs are approved for IBD, peripheral arthritis, PsA or axSpA. Abbreviations: axSpA, axial SpA; bid, twice daily; COX, cyclooxygenase; IBD, inflammatory bowel disease; IL, interleukin; IV, intravenous; JAK, Janus kinase; NSAID, non-steroidal anti-inflammatory drugs; od, once daily; PO, per os; SC, subcutaneous; SpA, spondyloarthritis; TNF, tumor necrosis factor.

## Data Availability

No new data were created or analyzed in this study.
